# Effect of wheat dextrin on corn flour extrusion characteristics

**DOI:** 10.1016/j.heliyon.2023.e21827

**Published:** 2023-11-04

**Authors:** Maxime Guéritte, Elia Dalle Fratte, Louise-Marie Van de Velde, Mia Eeckhout, Els Debonne

**Affiliations:** Research Unit of Cereal and Feed Technology, Department of Food Technology, Safety and Health, Faculty of Bioscience Engineering, Ghent University, Valentin Vaerwyckweg 1, 9000 Ghent, Belgium

**Keywords:** Breakfast cereals, Corn flour, Extrusion, Technical quality, Wheat dextrin

## Abstract

Wheat dextrin is a modified wheat starch, classified as water-soluble. This study investigated the effect of wheat dextrin as an ingredient in corn flour blends on extrusion characteristics. Blends were prepared at 0, 10 and 20 % fibre content. DOE was used to design experiments and investigate the effects of variables selected to be studied. Feed moisture content was set at 18–25 %, temperature at 110–150 °C and specific feeding load at 0.100–0.150kg/rev. Moisture content, water absorption and solubility indices, color, sectional expansion index, density, hardness, crispiness (work (W_c_) and number of spatial ruptures (N_sr_)) and specific mechanical energy were evaluated. A regression model was established using response surface methodology, and processing conditions for optimal quality were generated (e.g., WSI: 96.9 %, SME: 96.9 %, final MC: 93.9 %). Wheat dextrin solubility characteristics for moisture content, WAI and WSI were inconclusive, showing a high tendency to insoluble behavior. For expansion, lightness and SME characteristics depended on processing conditions, especially temperature. Crispness was highest at low MC (18.87 %) x high fiber content (20 %) (e.g., N_sr_: 1.2–1.5/mm), whereas values were the lowest at high MC (25.70 %) x low fiber content (0 %) (e.g., N_sr_: 0.5–0.7/mm). Optimal conditions were set at 12 % fiber content, 19 % feed moisture content, 130 °C and a specific feeding load of 0.146 kg/rev. This study showed that it is impossible to classify wheat dextrin as acting strictly according to soluble fiber characteristics based on extrudate characteristics.

## 1 Introduction

Food extrusion is widely applied in industrial cooking processes. It is a form of continuous processing that has been used since the 1930s in numerous food and feed applications, such as cereal-based and ready-to-eat snacks, e.g., baby foods, pasta products, ready-to-eat (RTE) cereals, modified starch from cereal sources, texturized vegetable proteins and other products [[1], [2], [3]]. The extrusion process includes high process temperatures, high pressures and high shear rates. The extrusion products - extrudates - are usually pre-ground and -conditioned raw materials that go through the feeding hopper in a heated stationary barrel. Water is added to the barrel, and the materials are pushed forward by tightly fitted screw(s), intensively mixed and increasingly heated by the heating elements [4]. These conditions result in several physical and chemical changes within the product, such as starch melting/-gelatinization, complex formation between amylose and lipids, protein- and other nutritional denaturations [5,6] and homogenization and melting of fats [7]. The extrudate exits the barrel by being pressed through a die, which usually expands and changes texture. It fixates due to the pressure- and temperature drop and steam release [6,8]. According to Singh et al. [9], consuming extrudates has many nutritional benefits. Sound effects are considered the destruction of anti-nutritional factors, starch gelatinization, increased soluble fiber content and reduced lipid oxidation. As such, it is believed that there is an improvement in the digestibility of proteins and starch [10].

On the other hand, depending on product composition and process conditions, a Maillard reaction between sugars and proteins reduces the nutritional value of amino acids, and the loss of heat-labile vitamins can be substantial due to heating processes [8,10]. Expanded extrusion products are second-generation snacks or directly expanded products (DEP) [1,11]. Ready-to-eat snacks (RTE's) are produced in the same way. They are the second most-produced extruded food products after pasta [1]. RTE and extruded snacks are manufactured from various cereal flours and starches [12]. If necessary, malt, fat, colorants, flavors, sugar(s), emulsifiers and/or salt is added [1,13]. The cooking of these snacks is generally done at low moisture levels (<20 %) and high temperatures (100–150 °C) [14,15]. These conditions ensure the puffing and expansion of the extrudates at the die. Generally, the extruded product will be dried to obtain a crispy or crunchy texture [1].

Indigestible carbohydrates as dietary fibers have caught the attention of many food scientists and technologists in the past decades, mainly due to their physiological benefits [[16], [17], [18], [19]]. Including dietary fibers in human diets provides health benefits such as a decrease of bowel disorders, risk of coronary diseases and type-II diabetes [16]. The nutritional effect of consuming dietary fibers is derived from the indigestibility and fermentation of their components in the large intestine [20]. These health benefits come from the physiological effects of consuming dietary fiber (e.g., the lowering of blood cholesterol and improvements in increased bowel function) and diabetes control by attenuation of blood glucose and insulin levels after meal consumption [21]. Dietary fiber also has a prebiotic activity by benefitting intestinal bacteria [16]. According to Mudgil and Barak [16], it is challenging to define dietary fibers, although the definition from the physiological and nutritional point of view has been accepted: ‘*polysaccharides and lignin that are undigested by the enzymes of the small intestine’*. The American Association of Cereal Chemists (AACC) defined dietary fiber in 2000 as *‘the edible parts of plant or analogous carbohydrates that are resistant to digestion and absorption in the human small intestine with complete or partial fermentation in the large intestine’* [22]. The components of dietary fibers are mainly polysaccharides. Fibers can be categorized according to their solubility, fermentability, physiological health benefits or cultivar sources [16]. The solubility of fiber is based on the ability for it to become - or not to become - soluble in water after extraction from plant cell walls [16,23,24] and influences the technical and nutritional characteristics of the processed fibers [25,26].

Considering the use of corn flour in producing cereal extrudates and the human need for high dietary fiber consumption, it was interesting to investigate the incorporation of fibers into RTE-cereal snacks. Several studies have explored the effects of fibers on the extruded product, with or without differentiating solubility characteristics and their functions in starchy extrusion [[27], [28], [29]]. It is known that generally, insoluble fibers have unwanted technical effects, and soluble fibers mainly do not influence end-product parameters [30,31]. Different results have been linked to the source of the investigated fibers [32].

Wheat dextrin *Nutriose FB06* is known for its prebiotic effects, low caloric value, and ability to decrease glycaemic responses [33,34]. In general, wheat dextrin is already widely used in the food industry due to its low viscosity and so develops the right consistency when added to water [35,36]. However, information found in the literature on adding chemically modified fibers to extrudates remains scarce. For example, the effect of *Nutriose FM06* on extrusion parameters has only been reported by Šárka and Smrčková [37] and Šárka and Smrčková [38], who studied the impact of physicochemical properties of the water-soluble fiber by controlling cooking variables in a single-screw laboratory extruder (e.g., resistant starch content and sensory properties of maize extrudates). There is little information on using *Nutriose FB06* in direct expansion extrusion. Two research questions have been formulated. First: “What are the effects of the process parameters (i.e., moisture content, temperature and specific feeding load) and the inclusion of wheat dextrin (*L Nutriose FB06*, Roquettes Frères) on extrudate quality parameters? Wheat dextrin effects have been attributed to soluble and insoluble fiber characteristics. Second: “What are the optimal process parameters for the extrusion of corn flour breakfast cereals, considering the fiber content?”. This second goal is process optimization. Depending on quality parameters that are deemed important in the consumption of extruded breakfast cereals, optimal values were defined to compare the test design results and predict optimal processing parameters.

## 2 Materials & methods

### 2.1 Raw materials

#### 2.1.1 Corn flour

The corn flour (CF) used for the production of the cereals was provided by N.V. Maselis (Roeselare, Belgium), called “Maizeflour”. The CF, delivered in 25 kg paper bags, had a 13.0 ± 0.3 % moisture content. This was validated by determination using the AACC International Method 44–15.02 (2010) [39], whereby moisture content is defined as a sample's weight loss when heated under specified conditions. Furthermore, the CF contained 1.5 % fat (15 % saturated, 30 % mono-unsaturated and 55 % poly-unsaturated), 7 % proteins, 78 % carbohydrates (of which 99 % starch), 3 % dietary fibers and 0,5 % ash and had a labeled nutritional value of 348 kcal/100 g (information provided by the manufacturer).

#### 2.1.2 Wheat dextrins

The added fibers were wheat dextrins (WD, chemically modified wheat starch), called “*L Nutriose FB 06*”. They were acquired from Oostvogels Logistics B.V. (Breda, the Netherlands), manufactured at Roquettes Frères (Lestrem, France). The wheat dextrins were delivered in paper bags. They had a fiber content of 86 % on a dry weight basis, 6 % reducing sugars, 0.03 % protein content and a moisture content of 4.6 % (information provided by the manufacturer). The amount of moisture was validated through the oven method: 4.6 ± 0.1 % (n = 5). Furthermore, they had an ash level of less than 0.10 %, and 90 % of the particles had a size smaller than 40 μm (information provided by the manufacturer).

### 2.2 Methods

#### 2.2.1 Experiment customised screening design

Design of Experiments (DOE) is a statistical method for designing test parameters to investigate multiple factors in an experiment [40]. Factors included in the design were fiber content (FC), feed moisture content (FMC), barrel temperature (T) and specific feeding load (SFL). A *Custom Design* (CD) was generated with JMP (SAS Institute, Buckinghamshire, UK), proposing 20 batches with factors set at three levels (low, medium, and high). FC-levels were set at 0, 10 and 20 %; FMC at 18.48, 21.58 and 24.67 %; T-levels at 110, 130 and 150 °C and SFL at 0.100, 0.125 and 0.150 kg/rev (screw revolution). An overview of all levels is shown in Table 1. To obtain the three FC-levels, three corresponding blends were made. The temperature levels as a factor were set in system parameters as the barrel temperature within the barrel (at the last section of the extrusion process). Multiple custom designs (CDs) were generated and evaluated with JMP. The terms that were assessed were the four factors and their interactions. The CDs were compared according to the power analysis of all terms, the prediction variance profile, the fraction of design space plot, a color map on confounding correlations between terms and design diagnostics evaluating D-, G-, A-efficiency and average variance of prediction (Supplementary data – figures A1, A2 and tables A1, A2).

**Table 1:** Ranges of independent variable levels and their coded values.

Independent		Levels		
**Variables**	**Code**	−1	0	1
Fibre Content (%)	x_1_	0	10	20
Feed Moisture Content (%)	x_2_	18.48	21.58	24.67
Barrel Temperature (°C)	x_3_	110	130	150
Specific Feeding Load (kg/rev)	x_4_	0.100	0.125	0.150

It was impossible to set exact values for powder flow and added water. Setting these parameters was only possible at integer levels. Therefore, It was impossible to generate extrudates with only three levels of moisture content in the barrel (FMC). The combination of three levels of water dosage (1.5, 2.5 or 3.5 kg/h, respectively the low, medium and high) and three levels of fiber content (FC, %) resulted in nine different values of real FMC-levels. The real FMC was calculated according to mass balance, using powder flow and added water values. Results of the calculations are given in Table 2, together with the final experimental setup. By altering the values, power design is changed. The initial design (old FMC-value) and final design (new FMC-value) were compared with JMP. The power analysis (Supplementary data – figure A3) shows that power has risen for all terms. This is expected as more variation in settings for one parameter raises the power level.

**Table 2:** Generated custom design with initial and recalculated moisture content; FC (fibre content), FMC (feed moisture content), T (temperature), SFL (specific feeding load).

Run	FC (%)	Intended FMC (%)^a^	Real FMC (%)**	T (°C)	SFL (kg/rev)
1	0	18.48	18.87	110	0.100
2	0	18.48	18.87	150	0.150
3	0	21.58	22.37	110	0.150
4	0	21.58	22.37	130	0.125
5	0	21.58	22.37	150	0.100
6	0	24.67	25.57	110	0.100
7	0	24.67	25.57	150	0.150
8	10	18.48	18.90	130	0.125
9	10	21.58	22.41	110	0.125
10	10	21.58	22.41	130	0.100
11	10	21.58	22.41	130	0.150
12	10	21.58	22.41	150	0.125
13	10	24.67	25.63	130	0.125
14	20	18.48	18.94	110	0.150
15	20	18.48	18.94	150	0.100
16	20	21.58	22.47	110	0.100
17	20	21.58	22.47	130	0.125
18	20	21.58	22.47	150	0.150
19	20	24.67	25.70	110	0.150
20	20	24.67	25.70	150	0.100

a Intended MC is given as the originally set MC.** The Real MC is based on calculations through the AACCI method 44–15.02 (2010).

#### 2.2.2 Sample preparation and extrusion cooking

##### 2.2.2.1 Preparation of the blends

Before extrusion, dietary fibers (wheat dextrin, WD) were added to the CF and blends were produced. Three different blends were obtained: blend 1 (100 % CF, 0 % WD), blend 2 (90 % CF, 10 % WD), blend 3 (80 % CF, 20 % WD). Due to the differences in MC between CF and WD, each blend initially has a different total MC. By calculating mass and water balances, the appropriate amount of water was added to blends 2 and 3 to get the same MC as blend 1 (100 % CF, with 13 % MC). The final MC was determined with the AACC Method 44–15.02 (2010) to confirm this method. Blend 1 contained an average MC of 13.02 ± 0.06 %; blend 2 resulted in an average MC of 12.85 ± 0.18 % and blend 3 resulted in an average MC of 13.13 ± 0.24 %. The blends were packed in closed plastic buckets and stored in a freezer at −22 °C for a maximum of 2 months.

##### 2.2.2.2 Extrusion cooking & drying

According to the generated CD, 20 batches with the three blends and different configuration settings were extruded in a co-rotating twin-screw extruder type BC45 (Clextral, France) at Food Pilot (ILVO, Melle, Belgium). The barrel had a length (L) of 1000 mm and a diameter (D) of 45 mm, which gives an L/D ratio of 22. It was built up in five modules. Modules 2 to 5 are heating elements (Supplementary data – figure A4). The die consisted of 3 holes with a 3 mm diameter. The extrudate was cut with a four-knife rotating cutter to obtain spherical shapes. Cutting the extrudate was not always possible because some batches produced too sticky samples. Therefore, they were not cut. Instead, long rod-shaped threads of the product were sampled. This was the case for sample runs 9, 10, 11, 14, 16, 19 and 20. The detailed processing characteristics of each run can be found in Table 2. After the production, the samples were kept in closed plastic buckets and transported to the Cereal lab at Campus Schoonmeersen (Ghent University, Ghent, Belgium), where they were stored at room temperature (20–23 °C). The samples were dried in a drying oven (Master Jerky 16, Klarstein; Berlin, Germany) to an MC_dry_ of 2–4 %. The dried product was conserved in closed and airtight plastic buckets at room temperature (20–23 °C) for a maximum of one week.

#### 2.2.3 Sample analyses

##### 2.2.3.1 Moisture content determination

Determining moisture content (MC) was performed according to the AACC Method 44–15.02 (2010). Samples were analyzed in one or two stages depending on the expected final moisture content. Sample runs with expected moisture lower than 18 % - i.e., 1, 2, 8, 14 and 15 - were analyzed using the 1-stage method. The other sample runs containing moisture content higher than 18 % - i.e., runs 3, 4, 5, 6, 7, 9, 10, 11, 12, 13, 16, 17, 18, 19 and 20 - were analyzed according to the 2-stage method. Initially, 40 g of extrudate sample was ground with Pulverisette 14 Rotary Speed Mill (Fritsch, Germany) with a 250 μm sieve at 8000 rpm. All ground samples were conserved in plastic coverable dishes. For all batches in the 1-stage method, 5 g of each ground sample was put in dishes (threefold) and dried in an oven for at least 4 h at 103 °C (Universal Oven UN55, Memmert GmbH). This was also done for CF, WD and the different CF-blends. Samples analyzed according to the 2-stage method were first oven-dried for 4 h at 60 °C, and the moisture loss during this procedure was calculated by weighing the samples before and after the oven drying. Subsequently, the samples were ground and oven-dried according to the 1-stage method. It is important to state that within this study, there were three different levels at which MC was determined/calculated: 1) feed moisture content (FMC: 18–25 %; *initial* MC); 2) moisture content of the extrudate at the die (MC_die_: 10.3–20.4 %; *final* MC); and 3) moisture content of the extrudate after drying (MC_dry_: 2–4 %). The terms *initial* and *final* refer to the start and end of the extrusion process.

##### 2.2.3.2 Sectional expansion and product density: image analysis

Sectional expansion and product density were calculated using image analysis [41]. For this, circa 30 pieces of each batch were scanned with 300 dpi (dots per inch) on an HP Scanjet 2400 (Hewlett-Packard Company; California, USA), using a dark background paper. The images were saved in ‘png-format’. These were analyzed with ImageJ Java (Oracle Corporation; California, USA). The scanned images were processed in the program so that the extrudates contrasted with the background. The dimensions of the scanned products were obtained in pixels and converted to centimeters. One pixel corresponded to 0.008467 cm. The new images were saved in “tif-format”. The data used for the calculation of the sectional expansion index (SEI) were the average minor (the smallest line passing through the centroid, in mm) and major diameter (the largest line passing through the centroid, in mm). The SEI was calculated as the quadratic ratio of the extruded product minor diameter (*D*_*e*_) to the diameter of the extruder die (*D*_*d*_), as shown in Eq. 1.

(1) $$SEI={\left(\frac{D_{e}}{D_{d}}\right)}^{2}$$

The scanned samples were weighed to calculate the product densities, and the individual particle volumes were calculated with the major and minor diameters obtained by ImageJ, resp. *Major* and *Minor*. Volumes were determined according to their shape (Eq. (2) and Eq. (3)). Spheres were considered ellipsoids, and the samples that were not cuttable by the extruder were broken into cylindrical shapes. The average density was computed by calculating the total weight per batch divided by the sum of all volumes (Eq. (4)).

(2) $$Volume\;ellipsoids\;\left({cm}^{3}\right)=\frac{4}{3}*\pi *Major*{Minor}^{2}$$

(3) $$Volume\;cylindrical\;shapes\;\left({cm}^{3}\right)=\pi *Major*{Minor}^{2}$$

(4) $$Density\;batch\;X\;\left(\frac{g}{{cm}^{3}}\right)=\frac{Total\;weight\;batch\;\left(g\right)}{\sum_{i=0}^{n}{Volume}_{i}\;\left({cm}^{3}\right)}$$

##### 2.2.3.3 Instrumental color analysis

The color of the extrudates was analyzed with a Konica Minolta CM-700d Spectrophotometer (Konica Minolta Sensing Americas, Inc.; Ramsey, USA). The undried samples were ground to particles smaller than 18 mesh (1 mm) beforehand. The granular samples were analyzed on an 8 mm area with an MAV lens, with D65 light coming from a pulsed xenon light source. For each batch, three samples were measured, and the CIE Lab lightness- (L*), redness- (a*) and yellowness values (b*) were obtained.

##### 2.2.3.4 Mechanical properties: texture analysis

The mechanical properties of the extrudates were analyzed with a TA-XT plus (Stable Micro Systems Ltd.; Surrey, UK). For each test, five samples per batch were analyzed. Two types of analyses were performed: puncturing and compression tests. The TA was equipped with a 5 kg loading cell and a P/2 N needle probe for the puncturing tests. The needle punctures the material at a speed of 1 mm/s, with a trigger force of 5 g and stops at 50 % strain. The post-test speed of the probe was likewise 1 mm/s. The acquisition of the plot was stopped at the trigger-return of the probe. All tests were performed at room temperature (20–23 °C). The compression test simulates hardness while biting. The TA had a 30 kg load cell and a cylindric probe. The probe had a flat end with a 1017.88 mm^2^ surface area (d = 36 mm). At the same speed as the previous test, the cylinder compressed the sample and stopped at 20 % strain. If compressed further, confounding data might be collected, as the sample sometimes breaks, and thus, the next force measured was the force applied to the broken piece. All tests performed with the TA provided a graph of the measured force (y-axis) over time (x-axis), generated with Exponent (Stable Micro Systems Ltd.; Surrey, UK). For the puncture tests, crispiness was calculated through the number of spatial ruptures (*N*_*sr*_) and hardness through the crispiness work (*W*_*c*_), according to Eqs. (5), (6)), respectively, as described by Pamies and Roudaut [42]. *N*_*0*_ represents the number of peaks counted in each graph generated, *d* the distance the probe traveled through the product and *F*_*m*_ the average puncturing force, which is the ratio of the measured area under the force-time curve (*A*, (m^2^)) to distance *d* (Eq. (7)). As each batch was measured five times, average values were generated for the analysis. The same amount of sample was analyzed as the other tests, five measurements were taken for each trial, and the same measurement conditions were kept. To count the peaks, a program was written in CoCalc (SageMath Inc., Washinton, USA), a platform for computable mathematics. The discrimination of peaks was done with the following settings: height = 0.05, relative height = 200, width = 0.01, threshold = none, distance = 40, prominence = 0.09.

(5) $$N_{sr}\;\left({mm}^{-1}\right)=\frac{N_{0}}{d}$$

(6) $$W_{c}\;\left(Nmm\right)=\frac{F_{m}}{N_{sr}}$$

(7) $$F_{m}\;\left(N\right)=\frac{A}{d}$$

##### 2.2.3.5 Water solubility and water absorption indices

Both the Water Absorption Index (WAI) and the Water Solubility Index (WSI) were determined according to the method of Anderson [43]. For each batch, 20 g of product was ground with Pulverisette 14 Rotary Speed Mill (Fritsch, Germany) consisting of a 200 μm sieve. Per batch, three samples of circa 2.5 g were weighed and mixed with 30 mL of distilled water in a centrifugal tube. The tubes were shaken on a Zeleny machine for 30 min (Kastenmüller Gmbh; Martinsried, Germany). Afterward, the tubes were centrifuged with a Sigma 3–18K (Sigma Laborzentrifugen GmbH; Osterode am Harz, Germany). The centrifuge worked at a relative centrifugal force of 9000 and centrifuged for 15 min at 30 °C. After centrifugation, the sediment was weighed for the calculation of the WAI according to Eq. (8). For the calculation of the WSI, the supernatant was oven-dried at 133 °C until all the water was evaporated. WSI was calculated according to Eq. (9).

(8) $$WAI=\frac{weight\;of\;sediment}{weight\;of\;dry\;solids}$$

(9) $$WSI=\frac{weight\;of\;dissolved\;solids\;in\;supernatans*100}{weight\;of\;dry\;solids}$$

#### 2.2.4 Statistical analysis

##### 2.2.4.1 Response surface method

The RSM was performed with JMP (SAS Institute, Buckinghamshire UK), where a model was fitted with terms as individual (*FC* (fiber content), *FMC* (feed moisture content), *T* (barrel temperature), *SFL* (specific feeding load)), squared factors (FC*FC, FMC*FMC, T*T, SFL*SFL) and cross-products (FC*FMC, FC*T, FC*SFL, FMC*T, FMC*SFL & T*SFL) for each dependent variable (responses from experimental data). This was done according to the following equation (Eq. 10):

(10) $$y_{i}=b_{0}+\sum_{i=1}^{4}b_{i}x_{i}+\sum_{i=1}^{4}\sum_{j=1}^{4}b_{ij}x_{i}x_{j}$$

In this equation, *y*_*i*_ is the dependent response variable; *b*_*0*_ and *b*_*i*_ are the regression coefficients for the constant and linear terms, respectively; *b*_*ij*_ is the coefficient of quadratic or cross-product terms; *x*_*i*_ and *x*_*j*_ are the coded values for the factors (independent variables), as given in Table 1. The dependent variables investigated were final MC (*MC*_*die*_); color parameters L*, a* and b*; *SEI* and *density*; *WA*I and *WSI*; mechanical properties *hardness* (force), crispiness work (*W*_*c*_) and crispiness number of spatial rupture (*N*_*sr*_) and *SME*. It is possible to fit the dependent variables together when fitting a model. As the color parameters L*, a* and b* are closely related, as well as the parameters for expansion interpretation *SEI* and *density*, they were fitted together. Afterward, each model was generated with a unique variable-specific summary effect, presenting LogWorth and p-values. LogWorth-values are the negative logarithmic transformation of p-values for visualizing purposes. The backward selection principle was applied, removing the effect with the highest p-value [44]. The selection was stopped when all remaining effects were significant (p < 0.05). Single-factor terms with no significance individually (p > 0.05) were kept when a squared or cross-product with this particular factor was found significant. Furthermore, JMP gives the possibility to convert the terms from LogWorth to False Discovery Rate (FDR) LogWorth, showing p-values with the FDR LogWorth for each model effect, calculated according to the technique described by Benjamini and Hochberg [45]. When significant differences were found - compared to the LogWorth p-values - the model was adjusted. P-value results, however, are always reported as a non-FDR LogWorth value. Once the model was designed by JMP, residuals by predicted plots were checked. Box-Cox or other logarithmic transformations are applied when a non-normal distribution is observed. The Box-Cox transformation was proposed by JMP, according to λ-value. This transformation is a powerful tool for normalizing data distribution [46,47]. The transformation was done according to the following equation (Eq. 11), where *x* represents the data and *λ* is the ‘power’ to which each data value is raised:

(11) $$x_{\lambda }^{\prime }=\frac{x^{\lambda }-1}{\lambda }$$

The R^2^ was given for each model. When this value was lower than 70 %, the model was considered to be weak, as the model explains less than 70 % of the data. Finally, parameter estimates were gathered for each model, predicting the dependent variable responses with a specific significance. Although significance levels were taken at p < 0.05, it was also stated when specific terms were found more significant (p < 0.01), highly significant (p < 0.001) or borderline (0.05 < p < 0.15). Significant RSM-model interactions (cross-product terms) were visualized in a three-dimensional response surface plot. This represents a response variable as a function of two interacting factors, showing a response plane predicting the behavior of the variables.

##### 2.2.4.2 Defining optimal parameters settings

The JMP “Profiler's Desirability Function” was executed to predict optimal parameter settings. The responses' color parameters, particle density, SEI, hardness and crispiness were assessed as parameters that can be optimized according to a specific reference. Honey Pops (Kellogg's) was chosen as a reference due to its similarity in the production process and raw materials. It must be stated that this is a product for market use, with the addition of salt, sugar, coating and other functional additives. This study is based on basic raw materials, without extra additives for consumer preferences and other product quality aspects. The results of these analyses were used as matching targets for the response goals within the model. Consequently, parameter settings at maximum desirability were obtained.

##### 2.2.4.3 Statistical significance determination

A multiple comparison analysis was performed using SPSS Statistics version 28 to assess significant differences among samples. Where the results were normally distributed, a Tukey test (homoscedasticity) or a Dunnett T3 test was used to describe the means with 95 % confidence (p = 0.05). A Dunn test for multiple comparisons was applied, preceded by a non-parametric one-way ANOVA test (Kruskal-Wallis) for non-normally distributed data. The results of this test are presented in Supplementary data (table A3).

## 3 Results

### 3.1 Response surface method

The results concerning the “Response Surface Method (RSM) Model” are described in this section for each end-product quality parameter. The exact results for each test per batch can be found in table A3(Supplementary data). Table 3 presents the results of the regression analysis. Coefficients for linear and quadratic effects, as well as cross-product interaction terms, are given. Significant cross-product interactions are visualized in response surface plots. The factors not shown in the plots are kept at centroid levels: FC at 10 %, (initial) FMC at 22.28 %, T at 130 °C and SFL at 0.125 kg/rev.

**Table 3:** Estimated regression coefficients of extruded corn flour breakfast cereals for responses final moisture content (final MC (MC_die_)), color measurements lightness (L^d^), redness (a^d^) and yellowness (b^d^), particle density (logarithmic form), water absorption index (WAI), water solubility index (WSI), hardness, crispiness and specific mechanical energy (SME).

Factor	Final MC	WAI	WSI (%)	L^d^	a^d^	b^d^	SEI	log(density) (kg/L)	Hardness (kg/s)	Crispiness W_c_ (Nmm)	Crispiness N_sr_ (/mm)	SME (kJ/kg)
Intercept	−0.0311^c^	−0.96^c^	68.35^a^	69.40^a^	5.68^a^	31.62^a^	60.95^a^	−5.86^a^	−12^c^	−400^c^	−2.06^a^	1201^a^
FC (x1)	0.0130^c^	−4.00^b^	61.94^a^	3.78^d^	−3.34^a^	−8.07^b^	8.97^c^	1.85^d^	5.4^c^	ns	0.11^c^	−650^a^
FC (x1x1)	ns	−26.03^c^	−377.78^b^	−27.72^c^	−22.06^c^	−116.77^a^	ns	ns	−520^b^	ns	ns	ns
MC (x2)	0.9329^a^	13.21^b^	−171.86^a^	75.62^a^	−29.62^a^	−14.04^c^	−90.15^a^	11.01^a^	45^c^	1487^b^	4.64^d^	−2071^a^
MC (x2x2)	ns	−490.12^d^	−3630.06^a^	−586.53^c^	385.45^b^	368.55^c^	−2912.80^b^	204.20^d^	ns	ns	ns	ns
T (x3)	−0.0005^a^	0.03^a^	0.07^d^	−0.03^d^	0.04^a^	0.00^c^	−0.10^b^	−0.01^b^	0^c^	ns	0.02^a^	−2^a^
T (x3x3)	ns	ns	ns	ns	ns	ns	ns	ns	ns	ns	ns	ns
SFL(x4)	0.3015^a^	3.56^c^	−118.09^a^	10.13^c^	−1.43^c^	17.40^c^	−139.05^a^	12.45^a^	2^c^	1354^b^	1.45^c^	−614^b^
SFL(x4x4)	ns	ns	ns	ns	ns	ns	ns	ns	8725^b^	ns	528.82^a^	ns
FC x MC (x1x2)	−1.9477^b^	ns	ns	ns	ns	ns	ns	ns	ns	ns	−93.61^a^	9421^a^
FC x T (x1x3)	ns	0.28^b^	0.07^c^	0.50^b^	−0.07^c^	−0.13^c^	−1.46^a^	0.04^c^	ns	ns	ns	ns
FC x SFL (x1x4)	ns	ns	ns	ns	ns	ns	−835.26^b^	27.86^c^	ns	ns	−71.10^b^	−12476^a^
MC x T (x2x3)	ns	0.19^c^	−4.22^b^	1.84^a^	−1.13^a^	−1.15^d^	ns	ns	3.9^b^	ns	0.42^b^	ns
MC x SFL (x2x4)	ns	−211.72^c^	3874.48^b^	−333.62^c^	175.90^c^	1416.03^a^	ns	ns	ns	ns	ns	27855^b^
T x SFL (x3x4)	ns	0.60^c^	5.24^b^	ns	ns	ns	6.65^a^	−0.51^b^	ns	ns	ns	60.881^a^
R^2^ (%)	93.3	91.3	96.9	90.2	96.3	80.9	87.6	84.0	59.6	47.1	91.8	96.9

Ns: Non-significant effects and interactions that are not taken into the model for a parameter.

FC (%) = fibre content (wheat dextrin).

MC (%) = initial moisture content (feed MC).

T (°C) = barrel temperature.

SFL (kg/rev) = specific feeding load.

R^2^ (%) = The proportion of the variance for each response explained by the regression model.

a Significant value at p < 0.01.

b Significant value at p < 0.05.

c Values in the model with insignificant levels (p > 0.05).

d Values in the model not significant at α = 0.05, with borderline significance (0.05 < p < 0.15).

#### 3.1.1 Final moisture content

**Fig. 1:**
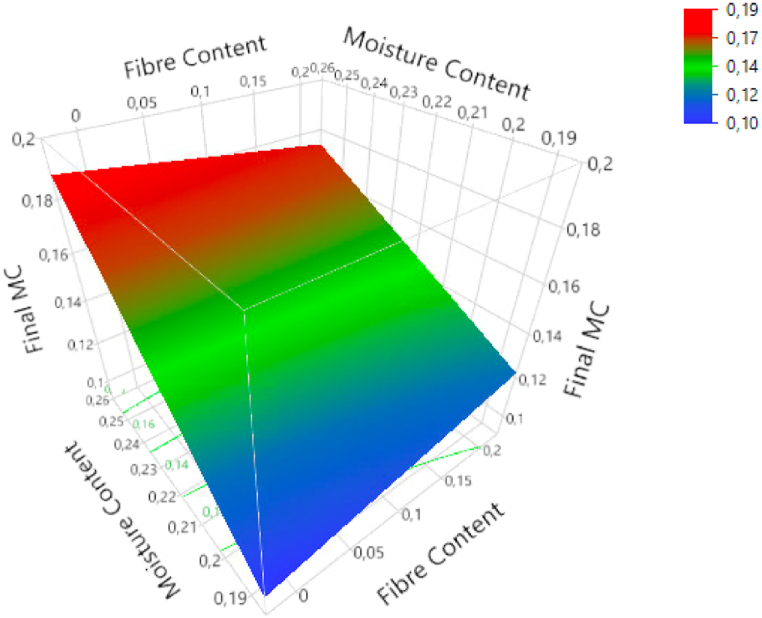
Response surface plot of final MC (MC_die_) of the interactions of initial MC (FMC) with FC.

Extrudate's final MC (MC_die_) responses ranged from 10.3 % to 20.4 %. A strong positive linear effect was recorded between the initial FMC and the final MC (p < 0.001). A negative linear effect for T, with high significance (p < 0.001), and a positive linear effect with SFL (p < 0.01) were observed. Although FC had no significant effect on the final MC (p = 0.555), there was an interaction between FC and initial MC on the final MC (p < 0.05). The initial MC (FMC) effect was the strongest at low FC (Fig. 1). Furthermore, FC had a positive effect at low FMC and a negative effect at high FMC.

#### 3.1.2 Water absorption index (WAI) and water solubility index (WSI)

**Fig. 2:**
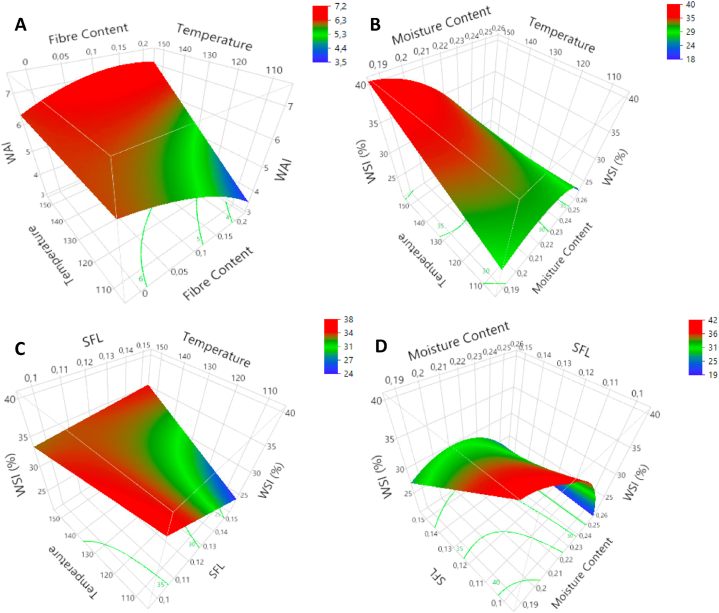
Response surface plots for WAI: T*FC (A), WSI: T*FMC (B); WSI: T*SFL (C) & WSI: SFL*FMC (D).

Responses for WAI ranged from 3.16 to 6.84 (Supplementary data – table A3). WAI responses were affected by three factors: FC, FMC and T. The T had a positive effect on WAI (p < 0.01). The FC had a negative linear effect on WAI (p < 0.05). Furthermore, FC was found to interact with T (p < 0.05). It can be seen in Fig. 2A that the negative effect of FC only applies at low T. The FMC had positive linear (p < 0.05) and borderline quadratic effects (p = 0.056). WAI increased with increasing FMC for low-to medium-levels (<23.5 %). After this plateau, WAI slightly decreased with increasing FMC (borderline significance: 0.05 < p < 0.15). The SFL did not affect WAI-values. WSI-analysis resulted in values within a range of 14.07–42.47 %. The FC had positive linear (p < 0.001) and negative quadratic effects on WSI (p < 0.05). Maximum WSI-values were found at medium to high FC-levels (±17.5 %). High FC resulted in low WSI-responses. The MC had a negative linear (p < 0.001) and quadratic effect (p < 0.01) on WSI. Contrary to WAI, WSI was influenced by SFL. A high SFL resulted in a low WSI (p < 0.01). The positive effect of T was not significant (p = 0.092). The T, however, interacted with FMC (p < 0.05) (Fig. 2B) and with SFL (p < 0.05) (Fig. 2C). At high T-levels, a high FMC resulted in a low WSI. Furthermore, the negative effect of SFL only applies at low T-levels. A positive effect of T was found at maximum SFL. Finally, an interaction was found between FMC and SFL (p < 0.05) (Fig. 2D). The negative effect of SFL on WSI only applies at low and medium FMC-levels.

#### 3.1.3 Color

**Fig. 3:**
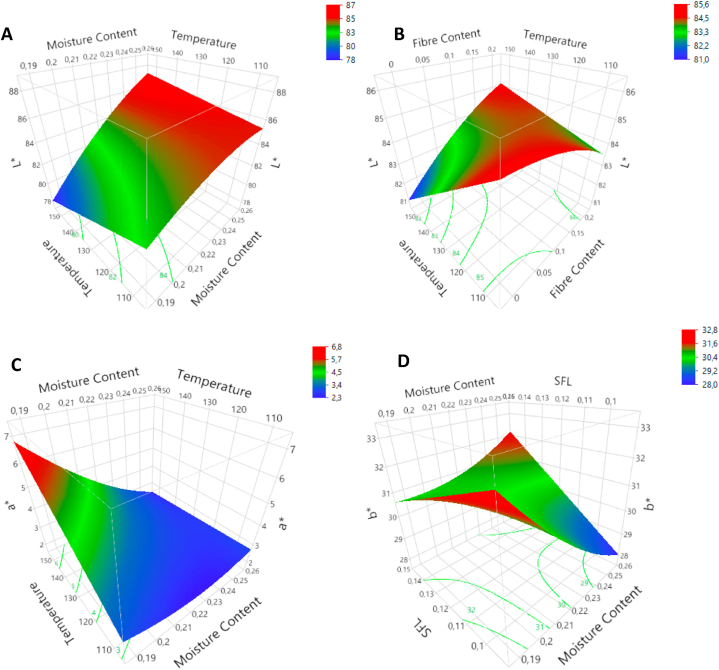
Response surface plots for colour parameters L: T*FMC (A); L: T*FC (B), a: T*FMC (C) and b: SFL*FMC (D). (For interpretation of the references to color in this figure legend, the reader is referred to the Web version of this article.)

All three responses were taken into the model together in the multiple regression analysis for the color parameters. The results can be found in table A3(Supplementary data). Responses are given for parameter L* for lightness (77.24–88.3), a* for redness (1.59–6.17) and b* for yellowness (24.34–31.49). Lightness was affected by FC, FMC and T. The FMC had a positive linear effect on lightness (p < 0.001). The negative effect of T was not significant (p = 0.071). An interaction between FMC and T was observed (p < 0.01), as shown in Fig. 3A. At low FMC, L* decreased with increasing T, while at high FMC, L* increased. The FC did not have significant linear or quadratic effects. However, it had an interaction with T (p < 0.05) (Fig. 3B). Without fibres, T decreased lightness, while at high FC-levels (20 %), lightness slightly increased with increasing T (p > 0.05). Redness was, likewise, affected by FC, FMC and T. The FC showed a negative linear effect (p < 0.01). For FMC, negative linear (p < 0.001) and positive quadratic effects (p < 0.05) were observed. As with lightness, an interaction (Fig. 3C) has been observed between FMC and T (p < 0.01). Yellowness was affected by FC, FMC and SFL. The FC decreased b* with linear (p < 0.05) and quadratic effects (p < 0.01). Although FMC and SFL neither had linear effects, they interacted with each other (p < 0.01). This interaction is shown in Fig. 3D. Yellowness decreased with increasing FMC at low SFL-levels and increased at high SFL-levels.

#### 3.1.4 Expansion volume and particle density

**Fig. 4:**
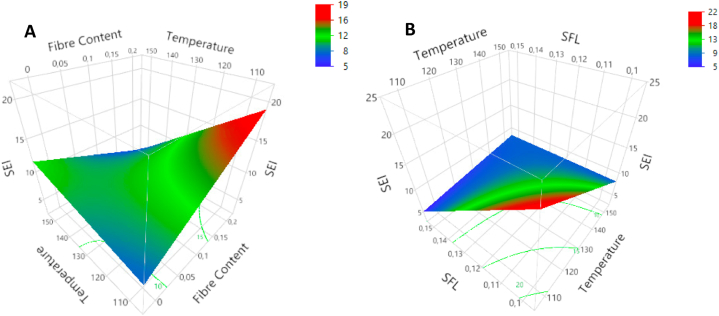
Response surface plots for sectional expansion index (SEI) as a function of T*FC (A) and T*SFL (B).

Sectional expansion indices (SEI) ranged from 5.1 to 26.7. The SEI was affected by all factors. The FMC had negative linear (p < 0.01) and negative quadratic effects (p < 0.05). The quadratic effect showed a slight increase in SEI for low FMC-values until a plateau at 21 % was reached (p > 0.05). For FMC-levels higher than 21 %, SEI decreased with increasing FMC. No significant individual effect was recorded for FC. An interaction between FC and T was observed for SEI (p < 0.01) (Fig. 4A). SEI increased with increasing T at low fiber levels, while at high FC (20 %), the effect was the opposite. SFL showed a negative linear effect (p < 0.001) and also interacted with T (p < 0.01) (Fig. 4B).

**Fig. 5:**
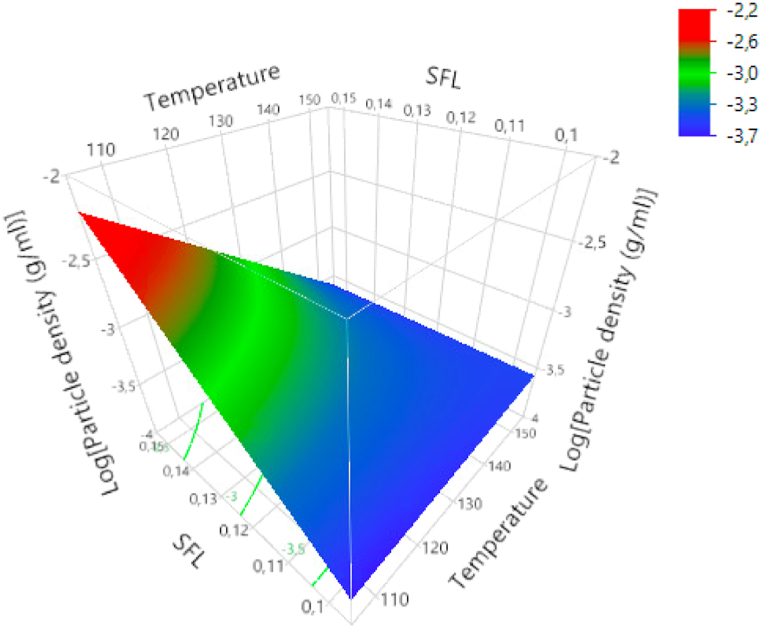
Response surface plots for density in logarithmic form as a function of T and SFL.

Responses for particle densities ranged from 0.0205 to 0.1682 kg/L. The logarithmic form was used to construct the regression analysis model. The residuals by predicted plots of both normal and logarithmic transformation are shown in figure A5 (Supplementary data). Positive linear effects on particle density were observed for FMC and SFL (p < 0.01) and a negative linear effect for T (p < 0.05). As for SEI, a quadratic effect was observed for MC, but without significance at α = 0.05 (p = 0.123). Likewise, FC was not found significant, but a positive effect was observed (p = 0.057). As seen in the surface plot of Fig. 5, T and SFL interacted with each other (p < 0.05). The results show that the positive linear effect of SFL on density weakens with increasing T-levels. The negative linear effect of T on density strongly depends on SFL.

#### 3.1.5 Mechanical properties

**Fig. 6:**
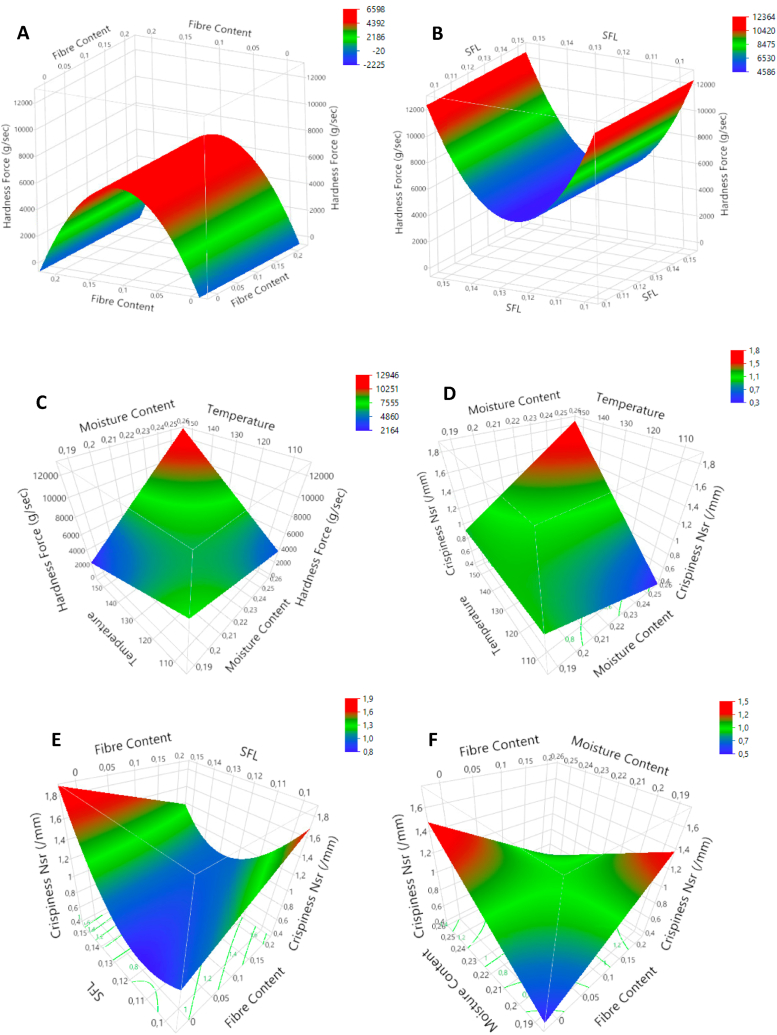
Response surface plots for hardness: FC*FC (A), SFL*SFL (B) & T*FMC (C) and crispness N_sr_: T*FMC (D), SFL*FC (E) & FMC*FC (F).

Hardness force (kg/s), crispiness work (W_c_, Nmm) and crispiness number of spatial ruptures (N_sr_, mm^−1^) are reported in table A3(Supplementary data). They range from 0.4 to 15.5 kg/s for hardness, 16 to 202.4 Nmm for W_c_ and 0.55 to 2.39 mm^−1^ for N_sr_. The impact of FC, FMC, T and SFL on these mechanical properties is presented in Table 3. The regression model for hardness force had a low R^2^-value (60 %) and recorded only three significant terms (all at p < 0.05). The FC was found to have a negative quadratic effect on hardness. Hardness increased with increasing FC until a plateau was reached at 10 % FC (Fig. 6A). The SFL had a positive quadratic effect on hardness (Fig. 6B). Both factors, FC and SFL, had no significant linear effect. Although MC and T have no individual effect, they do show an interaction, as shown in the surface plot in Fig. 6C. It must be stated that after FDR-correction, the p-values were high for the quadratic effect of FC (p = 0.074), the quadratic effect of SFL (p = 0.074) and for the interaction between MC and T (p = 0.113).

The crispiness work calculated from the puncture tests was affected by FMC and SFL (p < 0.05). Both factors had a positive effect on W_c_. No interactions nor quadratic effects were recorded. The number of cells measured, relative to the distance punctured (N_sr_), was affected by all factors. The T had a strictly positive linear effect (p < 0.001). The T interacted with FMC (p < 0.05). The surface plot is shown in Fig. 6D. The positive effect of FMC was not significant (p = 0.081), but the positive effect of T was the strongest at higher FMC-levels. A positive quadratic effect was recorded for SFL (p < 0.05). From the results, the lowest values for crispiness were found at medium SFL-levels (N_sr_ = 0.95 mm^−1^), high values at low SFL (N_sr_ = 1.15 mm^−1^) and even higher at high SFL-levels (N_sr_ = 1.4 mm^−1^). The FC interacted with SFL and FMC. The interaction between FC and SFL is shown in Fig. 6E (p < 0.05). At low SFL levels, FC positively affected crispiness; at high SFL, the effect was negative. The model recorded no individual effects for FMC and FC. An interaction was observed (Fig. 6F) (FMC*FC; p < 0.01). This interaction shows a positive effect of FMC at 0 % FC and a negative effect at 20 % FC and vice versa.

#### 3.1.6 Specific mechanical energy (SME)

**Fig. 7:**
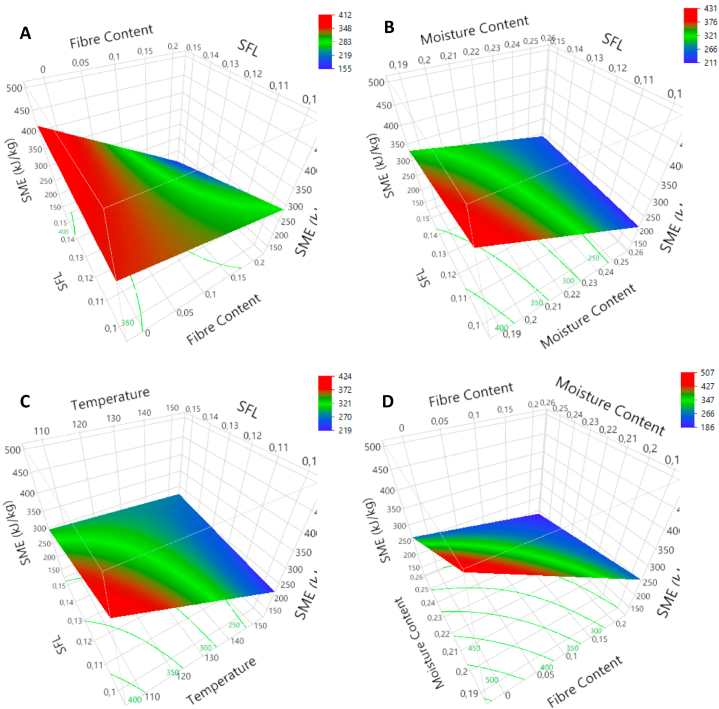
Response surface plots for specific mechanical energy (SME) as a function of SFL*FC (A), SFL*FMC (B), SFL*T (C) and FMC*FC (D).

SME was calculated, and the results ranged from 150 to 559 kJ/kg, as shown in table A3(Supplementary data). Negative linear effects with high significance were recorded for all factors individually (p < 0.001). No quadratic effects were observed. All three factors had an interaction with SFL. The negative effect of FC was the strongest at high SFL (p < 0.001) (Fig. 7A). The negative effect on SME was the strongest at low SFL for FMC (p < 0.05) (Fig. 7B) and T (p < 0.01) (Fig. 7C). The SFL, individually, had a negative effect on SME (p < 0.05). Finally, an interaction was observed between FC and FMC (p < 0.01) (Fig. 7D).

### 3.2 Optimal conditions definition

**Fig. 8:**
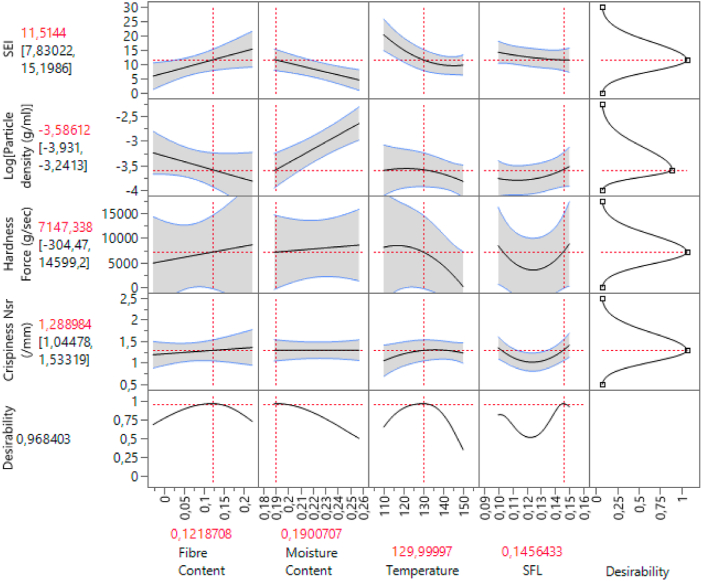
Prediction profiler for SEI, density (logarithmic transformation), hardness and crispiness, set at maximized desirability, based on input of fibre content, feed moisture content, temperature and specific feeding load.

The optimal conditions were set due to the analysis of particle density, SEI, hardness and crispiness of Honey Pops (Kellogg's). An average of 0.028 ± 0.004 kg/L (n = 25) particle density of the reference sample was measured, and an average SEI of 11 ± 1 (n = 25) was recorded. From 15 compressed samples, the mean value was calculated to be 3313 ± 1865 g/s as a reference for hardness. The results for crispiness (N_sr_) amounted to 1.7 ± 0.5 mm^−1^. These values were set as targets to be matched within the model, and optimal settings were acquired: FC = 12.2 %, FMC = 19.01 %, T = 130 °C, SFL = 0.146 kg/rev. It should be noted that particle density was incorporated with its logarithmic form (y = Log(x)). The setting was converted to y = −3.58612 kg/L. A total desirability of 96.84 % can be attained with these settings. Fig. 8 presents the prediction profiler, set at a level obtaining the highest desirability. Each response was recalculated and predicted with a certain amount of variance.

## 4 Discussion

### 4.1 Moisture content, water absorption and water solubility

The FC did not directly influence the final moisture content (MC_die_), but the interaction with initial MC (FMC) suggests that more moisture loss will occur at high FC. The interaction between FC and moisture loss at high FC can be explained by the decrease in glass temperature (Tg) when increasing insoluble fiber content [48]. As the glass temperature decreases, water has more time to evaporate from the product, thus reducing the water content [48]. However, it is more likely that blends with high FC-levels have more free water and thus result in more moisture loss. Wheat dextrin has a lower moisture content (4.7 %) than corn flour (13 %). Thus, more water was added to the blend. These findings agree with those reported by Brennan et al. [27], where adding insoluble fibers (bran) decreased final MC. This was also the case for guar (soluble fiber) [27]. According to Robin and Dubois [48], the increase of insoluble fiber content in raw materials used for extrusion reduces the starch content of the mixture and thus provides more free water in the melt.

WAI can be interpreted as a parameter of starch degradation, i.e., a high WAI indicates less suitability for starch degradation and, at the same time, more suitability for starch gelatinization. WAI content is positively correlated with melt viscosity [49]. WSI is an indicator of the degradation of starch and other molecular compounds [50]. The RSM-model predicts FC to have a negative effect on the WAI and a positive effect on WSI (Table 3). This could suggest that the fibers acted as insoluble fibers, as reported by Robin and Dubois [48] and Jin et al. [51]. Brennan et al. [27], however, reported that the addition of soluble and insoluble fibers resulted in less viscous melts within the barrel (with the exception of guar, which raised viscosity), suggesting a decrease in gelatinization for both types of fibres*.* The negative effect of fibres on WAI only occurred at low T-levels. This indicates that the effect of temperature on starch degradation was stronger, suggesting high starch degradation at all fiber content levels. This could also indicate that wheat dextrin degrades at high T, possibly reducing end-product fiber content. The negative quadratic effect of FC on WSI is in line with the findings of Jin et al. [51], suggesting wheat dextrin acted as an insoluble fiber.

### 4.2 Color properties

Fibers significantly made the extrudates less red and less yellow, which implies that the browning by the Maillard reaction could be neutralized when adding wheat dextrin [52]. This is expected for soluble fibers [28]. Wheat fibers made the extrudates lighter, but the significance was at the borderline. The replacement of corn flour with less colored material can result in less yellow/less dark extrudates since corn contains carotenoids that confer a yellow color to corn-based products [53]. When considering the interaction between FC and T, the darkening effect of T only applies at 0 % FC. By adding fibers, the darkening effect of temperature was nullified. This suggests that less starch degradation occurs when adding fibers, reducing the number of reducing sugars available for the non-enzymatic color reaction [26]. Furthermore, the impact of fiber addition on the browning potential and Maillard reaction of extruded corn snacks is dependent on the type of fiber used [52,54,55].

### 4.3 Expansion properties

When considering the effect of fiber content individually, it seems that sectional expansion was not affected. This could indicate that the fibers acted as water-soluble compounds. However, product density increased, indicating insoluble fiber behavior. The significance was at the borderline (p = 0.057). As Robin et al. [32] described, most studies concerning soluble fibers and their effect on expansion reported no changes. Some authors reported minor changes depending on the source of the fibers, the type of extruded raw material and their interactions [27,32,56]. In any case, it is widely accepted that the addition of insoluble fibers would decrease the SEI and increase bulk density [27,32,56]. Brennan et al. [27] reported that insoluble fibers induce bubble burst at the die exit, thus reducing expansion. Whereas a higher FC means a lower starch content and, therefore, less expansion [57].

When extruding at low T levels, FC had a positive effect on SEI, while at high temperatures, the FC affected SEI negatively. This suggests that the fibers acted as water-insoluble compounds, especially when working at high-temperature levels. This is in line with the positive effect of FC on density. SFL correlated well with die pressure but has been shown to affect expansion negatively and density positively. Both T and SFL gave unexpected results but had a significant interaction for both expansion and density. These results showed that to have an increase in expansion with increased T, a high SFL is needed. This explains the unexpected results, as more extreme processing conditions are needed for more predictable results. Once more, the behavior of the fibers is dependent on other extrusion processing conditions. These results highlighted the complexity of the interaction of fibers within extrusion and confirmed the uncertainty of the influence of - soluble - fibers as described by Robin et al. [32]. This complexity is created by the interactions of fibers with starch, differences in water sorption and plasticization behavior, and the physicochemical transformations they undergo during extrusion [32]. Treatment of (insoluble) fiber prior to extrusion can significantly improve its expansion and textural properties [30,32].

### 4.4 Mechanical properties

Adding insoluble fibers to a starch-based extrusion process increases the hardness [27,32,58]. The effects on crispiness are less conclusive as some researchers reported an increase in crispiness when adding insoluble fibers [27,59] and others a decrease [60,61]. There were no linear effects of fiber on hardness and crispiness. This could suggest that the fibers acted as soluble fibers. It is interesting to observe that there was, however, a quadratic effect of fibers on the hardness. Results showed that a maximum hardness was obtained at medium FC-levels, circa 10 % of added fibers. Menon et al. [36] found that beyond 10 % fortification, Nutriose FB06 did not favor the firm binding of freshly extruded sweet potato noodles. Nutriose FB06 might be forming a complex with gluten, which then encapsulates the starch granules, so they undergo only restricted swelling [36].

Furthermore, the interactions between FC and the factors MC and SFL are interesting to investigate. The positive effect of MC on crispiness (number of spatial ruptures) only occurred at 0 % FC, while at high fiber levels, the effect was the opposite. Consequently, a substantial interaction between wheat dextrin, starch and gluten is most likely. When observing the cross-product term of FMC and T, it is clear that a combination of both high FMC and high T-levels resulted in the crispiest extrudates. In contrast to crispiness, not many significant correlations were found in the model for predicting hardness. The absence of correlations between extrusion conditions and hardness should be interpreted with care, as a high variability on hardness force was measured, and the model had a low R^2^-value (60 %). This was also the case for crispiness work (R^2^ = 47 %), where FMC and SFL were the only significant factors. Crispiness work (W_c_) can be regarded as a measure of hardness but also as a value for crispiness. The increase in W_c_ with increasing FMC is in line with findings from Pamies and Roudaut [42].

### 4.5 Specific mechanical energy

Factors expected to influence SME are FMC, T, screw speed and feed rate [62]. The negative effect of FMC on SME confirmed the results of Colonna [11] and Ilo and Tomschik [62], as well as the negative effect of temperature, which has been reported by Gryczke [63]. The SFL, which has been reported to be correlated with SME by Unlu and Faller [64], had a negative effect on SME. It is interesting to observe that the addition of fibers decreased SME. This shows that the addition of fibers decreases the melts' total viscosity and thus decreases the specific energy needed for the extrusion process, a characteristic attributed to both soluble and insoluble dietary fibers in extrusion processes. This reduction can be attributed to water rehydration of the hydrocolloids [65]. It is interesting to consider the interactions of SFL with FC, T and FMC, which in turn were individually significant. The negative effect of fiber on SME was the strongest at high SFL-levels, suggesting that the decrease in viscosity with added wheat dextrin was high at high-pressure levels. Increasing SFL only decreased SME at low T and FMC-levels. This indicates that FMC and T have a stronger influence than SFL [66]. This is conclusive with the fact that the significance levels were highest for FMC and T (p < 0.001), compared to SFL (p < 0.05). Besides SME, which allows one to understand the energy consumption per unit product, knowing extruder efficiency would be beneficial to evaluate the overall machine performance and potential, which has an important industrial value [67].

### 4.6 Research limitations

Originally, feeding rate was going to be one of the four independent variables to investigate. However, it was not possible to set the extruder at the desired feeding rate. By manually performing calibration tests, it was discovered that the extruder's electro-mechanical system jumped between different working rates. The extruder engine was only working at integer values (i.e., 1, 2, 3 Hz, etc.). Hence, the engine was turning at fixed rates (i.e., 60 rpm, 120 rpm, 180 rpm, etc.). This was, however, not shown on the extruder's interface. When working with the prepared blends, the extruder worked at average feeding rates of 22, 36 and 66 kg/h. Consequently, it was decided to use SFL (kg/rev) as a factor. By adjusting the screw speed, exact SFL-values could be obtained. Thus, the feeding rate was not investigated. In the context of investigating the addition of chemically modified fibers and comparing this to other studies, it would have been more interesting to consider the feeding rate, which has been investigated by several other authors [9,47,50,62,68,69]. Several processing conditions resulted in extrudates that were not cuttable during the process. This was the case for three runs with 10 % fiber content (runs 9–11) and four runs with 20 % (runs 14, 16, 19–20). This shows that approximately half of the runs containing fibers produced an uncuttable product. In the performed data analysis, this was not a substantial problem. In practical industrial uses, this would, however, mean that these batches are not fit for production. Poor cutting is, according to Maskan and Altan [3], related to low dough viscosity and can be altered by changing the amount of amylose adjusting water content and shear conditions. It could be inherent to the wheat dextrin characteristics, as dextrin was found to be correlated with bread crumb stickiness [70].

The use of a cylindrical probe for the compression test was limited to the analysis of one sample particle at a time. Based on the literature and our results, the use of a Kramer Shear Cell (Stable Microsystems) is suggested, which is a bulk compression test [71,72]. In bulk, more particles are compressed, and variability will be low. Another research limitation was the lack of replicates. Within the limits of this study, the DOE was constrained to 20 different runs, including variations in FC, FMC, T and SFL. Each run was performed once. However, in order to reduce even more variability in the results, replicates and extensions of the number of experimental runs could be considered in the future.

## 5 Conclusion

The results show that it is not possible to classify wheat dextrin as acting strictly according to water-soluble fiber characteristics. It is, however, clear that its behavior is mostly dependent on other processing characteristics - in particular temperature - and that interaction with melt components occurs during processing. It is concluded that mechanisms behind the solubility characteristics are most likely related to the rheological properties of the melt and the interaction between the fibers and starch during the extrusion process. The investigation of melt viscosity and shear rate during extrusion is therefore deemed interesting. It can broaden knowledge in the domain of food extrusion and facilitate future processes and product optimization. In addition, investigating why certain samples were not cuttable is suggested. As has been shown in previous studies, the source of fibers also affects the influence on end-product parameters. It is, therefore, recommended that this research be repeated with chemically modified fibers from different sources or with other flour blends. Finally, researching the effect of other additives - e.g., salt, sugar, triglycerides - and their interactions with wheat dextrin in corn flour extrusion is recommended.

## Funding

This research did not receive any specific grant from funding agencies in the public, commercial, or not-for-profit sectors.

## Data availability statement

Data has not been deposited into a publicly available repository. Data can be made available on request.

## CRediT authorship contribution statement

**Maxime Guéritte:** Writing – original draft, Visualization, Methodology, Investigation, Formal analysis, Data curation, Conceptualization. **Elia Dalle Fratte:** Writing – original draft, Supervision, Methodology, Investigation, Formal analysis, Data curation, Conceptualization. **Louise-Marie Van de Velde:** Writing – review & editing. **Mia Eeckhout:** Supervision, Resources, Project administration. **Els Debonne:** Writing – review & editing, Writing – original draft, Supervision.

## Declaration of competing interest

The authors declare that they have no known competing financial interests or personal relationships that could have appeared to influence the work reported in this paper.
