# Supplementary material for: Effect of wheat dextrin on corn flour extrusion characteristics
Source: Heliyon. 2023 Nov 4;9(11):e21827. doi: 10.1016/j.heliyon.2023.e21827 (PMC10658250; doi:10.1016/j.heliyon.2023.e21827)
Supplement: Multimedia component 1 [file mmc1.docx]

**Supplementary data**

Custom Design Diagnostics (JMP, SAS Institute)

**Figure A1: Prediction variance profile**

**Table A1: Power analysis for custom design**

| **Term** | **Anticipated Coefficient** | **Power*** |
| --- | --- | --- |
| Intercept | 1 | 0,414 |
| Fibre Content | 1 | 0,865 |
| Moisture Content | 1 | 0,738 |
| Temperature | 1 | 0,861 |
| SFL | 1 | 0,859 |
| Fibre Content*Fibre Content | 1 | 0,288 |
| Fibre Content*Moisture Content | -1 | 0,644 |
| Moisture Content*Moisture Content | 1 | 0,432 |
| Fibre Content*Temperature | -1 | 0,809 |
| Moisture Content*Temperature | 1 | 0,644 |
| Temperature*Temperature | -1 | 0,281 |
| Fibre Content*SFL | 1 | 0,806 |
| Moisture Content*SFL | -1 | 0,644 |
| Temperature*SFL | 1 | 0,803 |
| SFL*SFL | -1 | 0,283 |

*Power analysis at α = 0,05

**Table A2: Design diagnostics**

| **Diagnosed Parameter** | **Result** |
| --- | --- |
| D-Efficiency (%) | 40,1287 |
| G-Efficiency (%) | 54,3343 |
| A-Efficiency (%) | 28,1681 |
| Average Variance of Prediction | 0,39452 |

**Figure A2: Colour map on term correlations**

**Figure A3: Power analysis of initial and definitive screening design (DSD)**

**
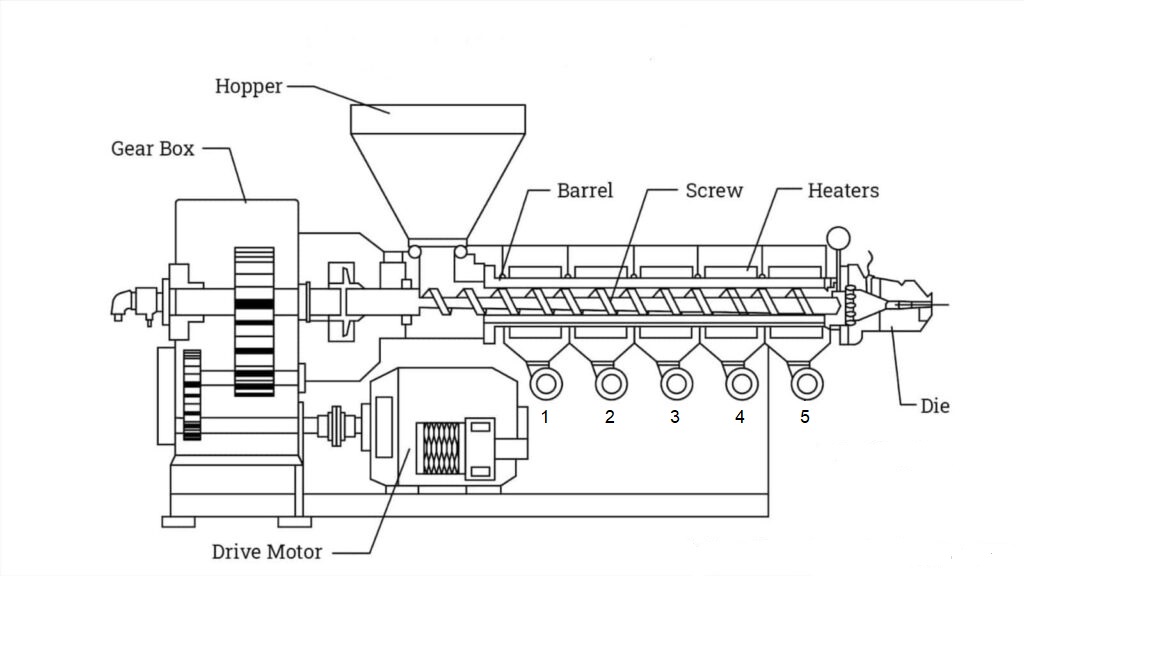
**

**Figure A4: Schematic overview of the extruder, composing of 5 modules (2-5 are heating elements; T_1_ = 20°C, T_2_= 50°C, T_3_= 95 – 110°C, T_4_ = 105-140°C; T_5_ = 110-150°C)**


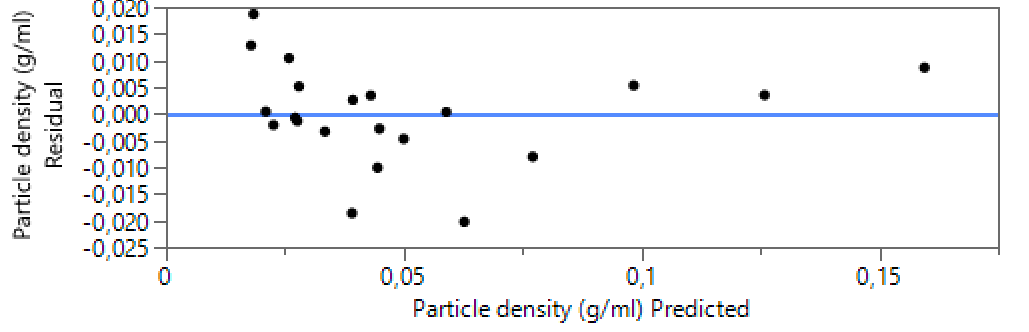


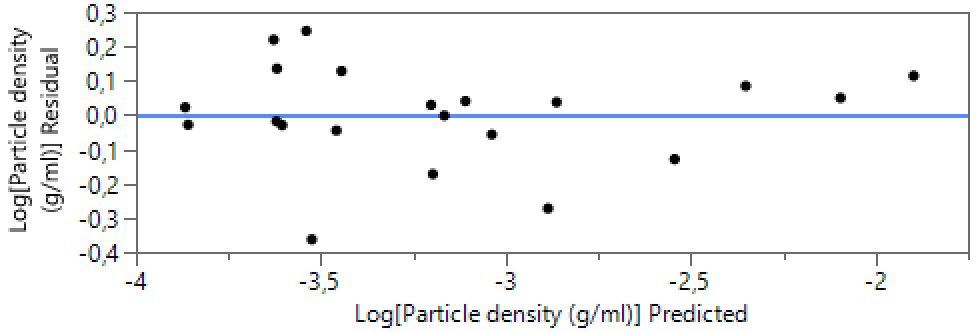


**Figure A5: Residual by predicted plots for particle density and its logarithmic transformation**

**Table A3: Overview of the principal responses for corn flour extrudates end-product parameters (mean ± std; n = 5)**

| **Run** | **Final MC** | **L*** | **a*** | **b*** | **SEI** | **Part. dens. (g/ml)** | **Log [Part. dens. (g/ml)]** | **WAI** | **WSI (%)** | **Hardness (g/s)** | **Crispness**  **W_c_ (Nmm)** | **Crispiness N_sr_ (/mm)** | **SME (kJ/kg)** |
| --- | --- | --- | --- | --- | --- | --- | --- | --- | --- | --- | --- | --- | --- |
| 1 | 0,103 | 82,2 ± 0,0^c^ | 3,3 ± 0,0^b^ | 30,2 ± 0,0^cd^ | 14,0 ± 0,5^f^ | 0,0263 ± 0,0042^ab^ | -3,638 | 5,0 ± 0,0^c^ | 26,8 ± 0,4^c^ | 2045 ± 300^bc^ | 48 ± 5^b^ | 0,64 ± 0,12^a^ | 558 |
| 2 | 0,104 | 77,2 ± 0,0^a^ | 6,2 ± 0,0^d^ | 30,4 ± 0,0^cd^ | 11,0 ± 0,3^e^ | 0,0205 ± 0,0032^a^ | -3,887 | 5,8 ± 0,0^d^ | 24,6 ± 0,6^b^ | 1374 ± 280^bc^ | 89 ± 9^c^ | 1,09 ± 0,16^cd^ | 436 |
| 3 | 0,161 | 84,8 ± 0,0^c^ | 2,7 ± 0,0^b^ | 29,5 ± 0,0^c^ | 5,1 ± 0,0^c^ | 0,0691 ± 0,0042^c^ | -2,672 | 6,0 ± 0,1^d^ | 17,0 ± 0,6^a^ | 1539 ± 320^bc^ | 131 ± 8^d^ | 1,35 ± 0,21^d^ | 422 |
| 4 | 0,144 | 84,2 ± 0,1^c^ | 3,0 ± 0,0^b^ | 29,6 ± 0,1^c^ | 7,6 ± 0,2^de^ | 0,0371 ± 0,0009^b^ | -3,294 | 6,0 ± 0,0^d^ | 19,5 ± 0,3^b^ | 2868 ± 530^c^ | 40 ± 7^b^ | 1,22 ± 0,18^d^ | 388 |
| 5 | 0,137 | 82,9 ± 0,0^c^ | 4,2 ± 0,0^b^ | 29,3 ± 0,0^c^ | 11 ± 0,4^e^ | 0,0205 ± 0,0005^ab^ | -3,887 | 6,2 ± 0,0^d^ | 22,7 ± 0,6^b^ | 3214 ± 743^c^ | 22 ± 3^ab^ | 1,15 ± 0,14^d^ | 285 |
| 6 | 0,187 | 86,1 ± 0,0^cd^ | 2,4 ± 0,0^b^ | 29,2 ± 0,0^c^ | 6,3 ± 0,4^d^ | 0,0465 ± 0,0050^b^ | -3,068 | 6,2 ± 0,0^d^ | 14,5 ± 0,3^a^ | 1733 ± 321^bc^ | 137 ± 12^d^ | 0,86 ± 0,08^bc^ | 299 |
| 7 | 0,173 | 85,0 ± 0,0c | 2,9 ± 0,0^b^ | 31,0 ± 0,0^d^ | 5,9 ± 0,6^d^ | 0,0419 ± 0,0042^b^ | -3,172 | 6,8 ± 0,0^e^ | 14,1 ± 0,2^a^ | 11820 ± 1465^f^ | 196 ± 23^ef^ | 2,39 ± 0,32^f^ | 278 |
| 8 | 0,121 | 82,5 ± 0,0^c^ | 4,5 ± 0,0^c^ | 31,5 ± 0,0^d^ | 9,6 ± 0,3^e^ | 0,0264 ± 0,0007^ab^ | -3,634 | 5,4 ± 0,2^cd^ | 34,6 ± 1,8^cd^ | 4936 ± 560^d^ | 56 ± 5^b^ | 0,97 ± 0,14^cd^ | 358 |
| 9 | 0,164 | 85,7 ± 0,0^cd^ | 2,4 ± 0,0^b^ | 28,7 ± 0,0^c^ | 16,0 ± 0,7^g^ | 0,0421 ± 0,0015^b^ | -3,168 | 6,1 ± 0,0^d^ | 28,5 ± 0,5^c^ | 4533 ± 720^d^ | 202 ± 2^f^ | 0,55 ± 0,09^a^ | 349 |
| 10 | 0,128 | 83,7 ± 0,0^c^ | 3,4 ± 0,0^b^ | 30,5 ± 0,0^cd^ | 11,0 ± 0,6^e^ | 0,0363 ± 0,0045^b^ | -3,316 | 5,7 ± 0,2^d^ | 33,9 ± 0,5^cd^ | 9065 ± 1520^e^ | 52 ± 7^b^ | 1,39 ± 0,06^d^ | 292 |
| 11 | 0,154 | 84,9 ± 0,0^c^ | 2,6 ± 0,0^b^ | 31,0 ± 0,0^d^ | 8,0 ± 0,2^de^ | 0,0593 ± 0,0063^c^ | -2,825 | 6,1 ± 0,0^d^ | 28,8 ± 0,5^c^ | 15466 ± 1893^g^ | 200 ± 24 | 1,54 ± 0,17^d^ | 276 |
| 12 | 0,147 | 82,6 ± 0,0^c^ | 4,1 ± 0,0^c^ | 30,9 ± 0,0^d^ | 8,6 ± 0,2^de^ | 0,0307 ± 0,0047^b^ | -3,483 | 6,3 ± 0,1^e^ | 27,9 ± 0,8^c^ | 3644 ± 450^c^ | 57 ± 3^b^ | 1,35 ± 0,09^d^ | 225 |
| 13 | 0,180 | 85,0 ± 0,0^c^ | 2,8 ± 0,0^b^ | 30,9 ± 0,0^d^ | 6,4 ± 0,2^d^ | 0,0425 ± 0,0052^b^ | -3,158 | 6,0 ± 0,1^d^ | 20,8 ± 1,0^b^ | 986 ± 145^b^ | 101 ± 14^c^ | 1,08 ± 0,18^cd^ | 243 |
| 14 | 0,141 | 82,4 ± 0,0^c^ | 2,7 ± 0,0^b^ | 28,1 ± 0,0^b^ | 6,0 ± 0,4^d^ | 0,1294 ± 0,0253^e^ | -2,045 | 3,2 ± 0,0^a^ | 21,0 ± 0,2^b^ | 3582 ± 362^c^ | - | - | 212 |
| 15 | 0,111 | 79,5 ± 0,0^b^ | 5,6 ± 0,0^d^ | 30,3 ± 0,0^cd^ | 11,0 ± 0,6^e^ | 0,0214 ± 0,0006^ab^ | -3,844 | 5,1 ± 0,1^c^ | 42,5 ± 1,0^e^ | 1846 ± 230^bc^ | 16 ± 3^a^ | 1,73 ± 0,09^e^ | 275 |
| 16 | 0,146 | 82,6 ± 0,0^c^ | 1,7 ± 0,0^a^ | 27,2 ± 0,0^b^ | 27,0 ± 0,9^h^ | 0,0301 ± 0,0015^b^ | -3,503 | 4,3 ± 0,0^b^ | 36,7 ± 0,3^d^ | 9208 ± 460^e^ | 92 ± 2^c^ | 0,94 ± 0,04^cd^ | 367 |
| 17 | 0,147 | 84,9 ± 0,0^c^ | 3,0 ± 0,0^b^ | 29,9 ± 0,0^cd^ | 7,9 ± 0,4^de^ | 0,0344 ± 0,0025^b^ | -3,370 | 5,9 ± 0,1^d^ | 32,4 ± 0,4^cd^ | 360 ± 56^a^ | 19 ± 3^ab^ | 0,71 ± 0,10^ab^ | 228 |
| 18 | 0,134 | 85,4 ± 0,0^c^ | 2,9 ± 0,0^b^ | 28,0 ± 0,0^b^ | 5,2 ± 0,3^c^ | 0,0453 ± 0,0063^b^ | -3,094 | - | - | 3348 ± 247^c^ | 131 ± 11^d^ | 1,57 ± 0,16^e^ | 215 |
| 19 | 0,204 | 85,1 ± 0,0^c^ | 2,4 ± 0,0^b^ | 30,1 ± 0,0^cd^ | 2,9 ± 0,1^b^ | 0,1682 ± 0,0036^f^ | -1,783 | 3,2 ± 0,4^a^ | 19,6 ± 0,8^b^ | 2410 ± 630^bc^ | - | - | 171 |
| 20 | 0,151 | 88,3 ± 0,0^d^ | 1,6 ± 0,0^a^ | 24,3 ± 0,0^a^ | 1,6 ± 0,0^a^ | 0,1037 ± 0,0028^d^ | -2,266 | - | - | 11775 ± 1700^f^ | 177 ± 12^e^ | 1,82 ± 0,14^e^ | 150 |

Superscripts with different letters indicate significantly different values (p < 0.05).
